# Domesticated LTR-Retrotransposon *gag*-Related Gene (*Gagr*) as a Member of the Stress Response Network in *Drosophila*

**DOI:** 10.3390/life12030364

**Published:** 2022-03-03

**Authors:** Lidia Nefedova, Alexey Gigin, Alexander Kim

**Affiliations:** 1 Department of Genetics, M.V. Lomonosov Moscow State University, Leninskie Gory, 1, 119991 Moscow, Russia; trex74749z@gmail.com (A.G.); aikim57@mail.ru (A.K.); 2 Faculty of Biology, Shenzhen MSU-BIT University, Longgang District, Shenzhen 518172, China

**Keywords:** molecular domestication, retroelements, *Drosophila*, evolution, stress response

## Abstract

The most important sources of new components of genomes are transposable elements, which can occupy more than half of the nucleotide sequence of the genome in higher eukaryotes. Among the mobile components of a genome, a special place is occupied by retroelements, which are similar to retroviruses in terms of their mechanisms of integration into a host genome. The process of positive selection of certain sequences of transposable elements and retroviruses in a host genome is commonly called molecular domestication. There are many examples of evolutionary adaptations of *gag* (retroviral capsid) sequences as new regulatory sequences of different genes in mammals, where domesticated *gag* genes take part in placenta functioning and embryogenesis, regulation of apoptosis, hematopoiesis, and metabolism. The only *gag*-related gene has been found in the *Drosophila* genome—*Gagr*. According to the large-scale transcriptomic and proteomic analysis data, the *Gagr* gene in *D. melanogaster* is a component of the protein complex involved in the stress response. In this work, we consider the evolutionary processes that led to the formation of a new function of the domesticated *gag* gene and its adaptation to participation in the stress response. We discuss the possible functional role of the Gagr as part of the complex with its partners in *Drosophila*, and the pathway of evolution of proteins of the complex in eukaryotes to determine the benefit of the domesticated retroelement *gag* gene.

## 1. Introduction

The term “molecular domestication” was first proposed by Wolfgang Miller in 1997 to describe the phenomenon of the adaptation of the sequences of mobile elements by the organism for its benefit [1]. The molecular domestication of retrotransposons and retroviruses and their distinct sequences can play a significant role in the formation of new gene families that define the configuration of modern eukaryotic taxa. The role of domesticated retroelement genes in mammals has been studied in detail. For all three genes of LTR retroelements and retoroviruses, *gag*, *pol*, and *env*, domesticated homologs with functions beneficial for the host organism have been discovered. The greatest variety of such genes has been found for homologs of the capsid gene, *gag.* Several gene families with a retroviral *gag* origin (*PNMA*, *Mart*, and *SIRH*) have been found, which play an important role in placenta functioning and embryogenesis, regulation of apoptosis, hematopoiesis, metabolism, etc. [2,3,4,5,6]. A number of other domesticated retroelement *gag* genes may be involved in protection against retroviruses (for example, the *Fv1* gene in mice) [7,8,9]. Domesticated retrotransposon *gag* sequences have also been found in vertebrates; some of them occur simultaneously in many genes. For example, sequences coding for the SCAN domain is specific for many transcriptional factors found in vertebrates [10].

Cases of molecular domestication of retroelements and their sequences in invertebrates have been investigated much less often than in vertebrates. Most studies on LTR retrotransposons have been done on *Drosophila melanogaster*. The *D. melanogaster* genome contains many known families of LTR retroelements (including the most representative family, Gypsy, which contains representatives classified as retroviruses) [11]. Many studies have been dedicated to investigating LTR retroelement transposition and their influence on the *D. melanogaster* genome, but there are little data on the molecular domestication of retroelements. Currently, the only domesticated retroelement *gag* gene, *Gagr* (homolog of the *gag* gene of the Gypsy group of LTR retroelements), is known, and its functions have been partially characterized [12]. The *Gagr* gene is possibly associated with the origin of new functions and the involvement in stress response in *Drosophila* species [13]. *Gagr* expression is activated in response to the induction by bacterial lipopolysaccharides in S2 cells, and this activation depends on the regulators of the MAPK/JNK stress signaling pathways *Tak1*, *hep* and *bsk* [14]. *Gagr* expression increases significantly after intraabdominal injection of DCV viruses (*Drosophila* C virus), FHV (Flock House virus), and SINV (Sindbis virus) [15]. Establishing a correlation between the variability in sequences of the *Gagr* gene homologs in a number of *Drosophila* species and the modification of its functions will make it possible to better understand the processes of molecular domestication.

In previous studies, we found that the stress-induced activation of the *Gagr* gene is controlled by potential regulators involved in endoplasmic reticulum (ER) stress. The *Gagr* gene is involved in a cellular oxidative stress response but is not activated under mitochondrial stress [13]. The product of the *Gagr* gene has membrane localization [12].

**Figure 1:**
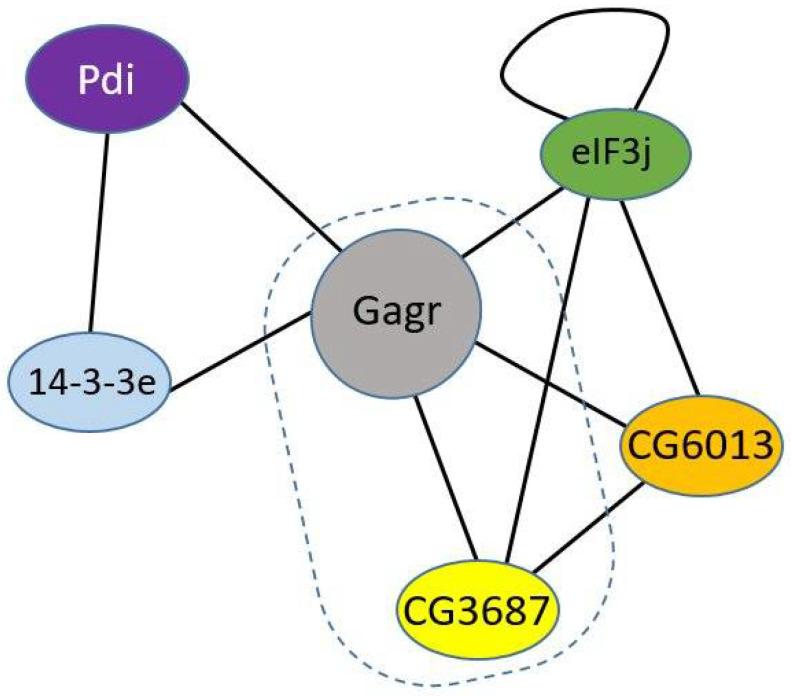
Physical protein–protein interactions for the Gagr were obtained by experimental results [16]. 14-3-3e, Pdi, eIF3j and CG6013 are conservative components of the complex, Gagr and CG3687 are components arising in insects (circled with a dotted line).

Valuable information for the prediction of the function of the Gagr protein can be found in Guruharsha et al. [16], which contains data on a number of protein–protein interactions and the composition of protein complexes. In this work, high-throughput mass spectrometry of protein complexes was used. Protein complexes were purified using antibodies for the universal epitope of the transiently expressed protein, which was a part of the complex. This protein was expressed in *D. melanogaster* S2R+ cells [16]. The following protein partners were found for the Gagr protein—products of the genes *14-3-3epsilon*, *Pdi*, *eIF3j*, *CG6013*, and *CG3687* (Figure 1); four of them (*14-3-3epsilon*, *Pdi*, *eIF3j*, and *CG6013*) have highly conserved functions in eukaryotes.

In this study, we discuss the possible functional role of the Gagr as part of the complex with its partners in *Drosophila*, and the pathway of evolution of proteins of the complex in eukaryotes to determine the benefit of the domesticated retroelement *gag* gene.

## 2. Components of the Complex That Are Conserved in Eukaryotes

### 2.1. 14-3-3e

The 14-3-3e protein is a conserved multifunctional protein of the 14-3-3 family of regulatory proteins found in all eukaryotes [17,18,19,20,21,22,23]. This protein, as a rule, serves as an adapter for protein–protein interactions, providing close contact between interacting molecules, or as an inhibitor that suppresses the activity of a bound protein [18]. All 14-3-3 proteins can bind a multitude of functionally diverse signaling proteins, including kinases, phosphatases, and transmembrane receptors; more than 200 signaling proteins have been reported as 14-3-3 ligands [19]. There are common recognition motifs for 14-3-3 proteins that contain a phosphorylated serine or threonine residue, although binding to non-phosphorylated ligands has also been reported [19]. It is assumed that 14-3-3e is phylogenetically the most ancient 14-3-3-protein, which later gave rise to several paralogs [20].

Homologs of 14-3-3e are well studied in *Arabidopsis thaliana* [21,22] and yeast [23]. The most characteristic function of yeast homologs of 14-3-3 is cell cycle control. Thus, the 14-3-3 homologue of *Schizosaccharomyces pombe*, Rad25, is necessary for the correct passage of the G2-M checkpoint; its absence leads to premature initiation of mitosis (meiosis) [23]. Rad25 is also involved in the DNA-damage checkpoint and bound to both the N-terminal and the C-terminal domains of mitogen-activated protein kinase (MAPK) Byr2 [24]. Rad25 can control the passage of G2-M in at least two ways: by suppressing Byr2 activity and by interacting with Ago proteins, leading to the interception of mitosis-stimulating phosphatases [25,26]. Homologous proteins in *Saccharomyces cerevisiae* (BMH1 and BMH2) have been implicated both in Ras/MAPK signaling and rapamycin-sensitive signaling [18,27,28]. The 14-3-3 proteins are known to bind transcription factors in a phosphorylation-dependent manner and modulate their subcellular localization and thus their activity [29,30].

In *Drosophila* as well as in humans, 14-3-3e is an important factor in the regulation of the MAPK and hippo signaling pathways, as well as the cell cycle, apoptosis, and a number of metabolic processes [20,31]

### 2.2. Pdi 

Pdi (disulfide isomerase) is a conserved redox-sensitive eukaryotic chaperone [32]. Most often, Pdi is localized in the ER as part of a tetrameric complex with prolyl-4-hydroxylase (a collagen-processing enzyme) [33], but it can also be present in the cytoplasm and on cytoskeletal elements [34]. 

Pdi homologs in *S. cerevisiae* are localized only in the ER [35]. The Pdi of *A. thaliana* is localized in the ER, as well as in the cytosol and in the nucleus [36]. In human, the PDI gene family contains 21 members, varying in domain composition, molecular weight, tissue expression, and cellular processing; loss of Pdi activity has been associated with pathogenesis, most commonly related to the unfolded protein response (UPR) [32]. In *D. melanogaster*, Pdi is located in several cellular components, including the fusome, rough endoplasmic reticulum and spindle envelope [37,38]. Pdi of *D. melanogaster* belongs to a family of 24 thioredoxin domain-containing proteins, and is the closest paralog in the ERp60 protein. According to an analysis [16], Pdi interacts with approximately 80 proteins in *Drosophila*. It has been shown that only the dimer is able to phosphorylate, while some experiments have suggested that within the ER, the phosphorylated form of Pdi is mainly mobilized in larger sized oligomers. Thus, a possible role for this phosphorylation may be to modulate the association of Pdi with its different partners [20,38]. 

Pdi is induced during ER stress and serves as a cellular defense against protein misfolding via its chaperone activity [39]. It is responsible for the isomerization, formation, and rearrangement of protein disulfide bonds, thereby providing another mechanism by which native protein conformation is maintained [40]. The accumulation of misfolded proteins within the ER activates the UPR. The UPR aims to reduce the load of unfolded proteins by increasing the curvature of the ER, reducing protein synthesis, and by the induction of Pdi and other chaperones to further increase the protein folding capacity [40,41]. This is achieved by activation of sensor ER proteins inositol requiring enzyme-1(IRE-1), protein kinase RNA-like ER kinase (PERK), and activating transcription factor kinase 6 (ATF6), which subsequently activate UPR signaling pathways [42]. Pdi can also activate the transcription factors NF-kB and AP-1, thus promoting their binding to DNA [41,43]. Presumably, Pdi can be involved in stress-dependent cascades, causing changes in the redox potential in the cell, probably not only in the ER and not directly, but through some other primary redox sensor [34,42,44].

### 2.3. eIF3j 

eIF3j, a highly conserved eukaryotic protein, is one of the subunits of the eukaryotic translation initiation factor 3. The main function of this complex is to ensure the attachment of the small subunit of the ribosome to mRNA. It has been shown that the eIF3j protein is required for 18S rRNA maturation [45], for recruitment of other eIF3 components to the small ribosome subunit under normal conditions [46], and for dependent on internal ribosome entry site (IRES) translation, which occurs under conditions of acute cellular stress [47]. As a rule, switching to IRES-dependent translation occurs upon suppression (in the case of stress) or inhibition (in the presence of a long and/or highly structured 5’-UTR in mRNA) of the “canonical” cap-dependent pathway [48]. Switching usually requires the presence of specific stimulus factors (ITAF) and some standard initiation factors (most often eIF4a and eIF3j, as well as other components of eIF3, but not eIF4e and eIF4g) [48,49]. The eIF-3 complex specifically targets and initiates translation of a subset of mRNAs involved in cell proliferation [48]. During translation initiation, eIF3j remains loosely bound to other eIF3 components and the small ribosome subunit and does not contact directly with IRES. Nevertheless, the presence of eIF3j is important both for maintaining the stability of the entire complex and for its key conformational rearrangements [50].

### 2.4. CG6013 

In *D. melanogaster*, the *CG6013* gene is annotated as a gene with an unknown function, which has homology with the human *CCDC124* gene. At the amino acid level, CG6013 has all the typical features of CCDC124 (N-terminal DNA/RNA-binding domain, C-terminal HMG box, potentially functional NLS and NES signal sequences) [51].

Orthologs of the *CCDC124* gene are known in many species from various eukaryotic taxa; however, their functions have been partially studied only in yeasts [51], humans [52], and mice [51]. In the yeast *S. pombe*, the product of the orthologous *Oxs1* gene is a cofactor of the transcription factor (TF) Pap1 (AP-1-like transcription factor) in the Pap1/Oxs1 signaling pathway [51]. This pathway is activated in response to very specific events: disulfide bond stress (the formation of excess crosslinks of thiol groups in a protein molecule due to their oxidation or binding to heavy metals) [51]. In yeast, this pathway does not respond to peroxides (although Pap1 itself, as a redox-dependent TF, does). Fission yeast responds to high concentrations of peroxides by activating the MAPK cascade, which includes elements of the JNK pathway (as such, the JNK pathway is absent in yeast, and the yeast homologue of JNK2, Sty1, is a part of the MAPK pathway and activates Atf1) [51,53]. 

Oxs1 has several activities: DNA-binding (landing on promoters) and polypeptide-binding (interaction with Crm1/Xpo1 exportin). Functionally, Oxs1 presumably behaves as a cofactor for Pap1, enhancing its activity as a TF and changing its specificity (allowing the activation of new target genes) [51]. The development of a Pap1-dependent stress response depends on further export of Oxs1 from the nucleus; this may be due to the suppression of Pap1 export due to the predominant capture of Oxs1 molecules by exportin-1 (possibly, during the exit of mRNAs with which Oxs1 can interact from the nucleus; their export just depends on Xpo1). Yeast carrying the Oxs1 gene with a defective NES did not develop a stress response [51]. A defect in NES Pap1, on the contrary, led to the development of the reaction since Pap1 was no longer removed from the nucleus by exportin-1 [51,53,54].

In the yeast *S. cerevisiae*, the Lso2 protein, a homologue of the N-terminal region of CCDC124, has been described [55]. This protein in yeast is presumably involved in the (re)initiation of translation after various stress reactions (protein or carbohydrate starvation, osmotic shock). It is known that Lso2 is able to bind to the active center of the ribosome, stabilizing the particle itself in the absence of mRNA and tRNA. When restoring translational activity, Lso2 is absolutely necessary at all stages; in Lso2-defective yeast, translation deregulation was observed (delays or cessation of initiation, premature start of elongation, often assembly of “empty” particles) [55]. The region of the protein that binds to the ribosome corresponds to the coiled coil domain. Human CCDC124 has a domain that is very similar in amino acid composition and retains ribosome-binding activity, which has been shown in transgenic yeast carrying the human gene instead of Lso2 [52,55]. Thus, Lso2 facilitates rapid translation reactivation by stabilizing the recycling-competent state of inactive ribosomes.

The human CCDC124 protein has been found to have mRNA-binding activity [56]. Moreover, in experiments, the binding of CCDC124 to the ADARB1, PRKRA, and EFTUD2 proteins and the U1 complex (ADARB1 is an RNA-processing enzyme, PRKRA is a kinase activated by dsRNA, and EFTUD2 and U1 are components of the spliceosome) [57,58], as well as an interaction with a large subunit of the ribosome, exportin-1, and ribosome-specific kinase (as in the yeast *S. cerevisiae*) have been demonstrated [59]. These data may indicate a close association of CCDC124 with the translation apparatus (possibly also with the splicing apparatus). Given the homology with Lso2, it can be assumed that CCDC124 has functions associated with stress-dependent (re)activation of the translational apparatus. The possibility of nuclear localization of CCDC124 has not been shown, but its NLS and NES are similar to those of Oxs1 [51]. It is known that CCDC124 is localized on elements of the cytoskeleton (actin filaments), on centrioles, and in the cytoplasm [60]. Its interactions in vitro and in vivo with ribosomal proteins and ribosomes are also described [55]. At least in yeast, the interchangeability of human and mouse Oxs1 and CCDC124, as well as that of *Drosophila* CG6013 and a homologue in *A. thaliana* paired with TF Pap1, have been shown. In turn, Pap1 can be replaced by TF bZip10 from *A. thaliana* [51].

## 3. Components of the Complex of Interactions That Have Arisen in Insects 

### 3.1. CG3687

CG3687 is a poorly studied *D. melanogaster* protein consisting of two domains: an N-terminal domain with unknown functions and a C-terminal LysM domain (according to BLAST analysis). Only five publications mention the gene in large-scale studies. One of them indicates *CG3687* as a gene related to muscle morphogenesis and function in *Drosphila* [61]. However, there are no research papers aimed at studying of the *CG3687* gene function.Thus, the data below was obtained during the analysis of the information presented in the FlyBase database [62] and through the BLAST search [63] as well as by use of several programs predicting domains and important sites in proteins.

**Figure 2:**
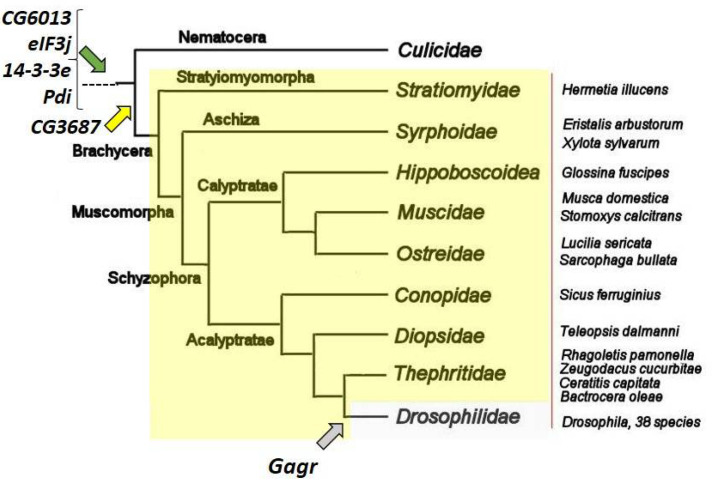
Phylogenetic tree illustrating the time of inclusion of the *CG3687* and *Gagr* homologs into the core complex of interactions of the *CG6013*, *eIF3j*, *14-3-3e*, and *Pdi* genes. The *CG6013*, *eIF3j*, *14-3-3e*, and *Pdi* homologs (green arrow) have conservative structures and functions for eukaryotes. The *Gagr* gene (gray arrow) has homologs only in the *Drosophilidae* genomes (grey box). The *CG3687* gene (yellow arrow) has homologs in genomes of flies within the Brachycera suborder (yellow box): protein BLAST analysis [63] finds similar proteins in genomes of the 38 *Drosophilidae* species and 14 other species of the other families (shown near the red line). Phylogenetic tree is modified after [64].

Homologues of the N-terminal domain have been found not only in Schizophora, but also in Syrphoidea (sister group) and Stratiomyomorpha (basal Brachicera) (Figure 2). The C-terminal domain of these homologues is very different from that of LysM. Complete homologues of *CG3687* have been found in representatives of the *Tephritidae* family and in *Musca domestica*. In all studied species, the *CG3687* gene retains its structure (two exons that do not correspond to the division into domains in the protein product).

The origin of the *CG3687* gene is not entirely clear. At least in *Drosophila* and *Tephritidae*, the *CG3687* gene (or a homologue of its N-terminal region) belongs to the same group of “neighbor” genes, including *HLF* (TF bZIP), *MFS* (presumably transport protein), *TFS3* (transferrin 3), *CG15701* (presumably a dynein adapter), *krimp* (TUDOR protein), and *Nup75/85* (nuclear pore component). In the flies of the Muscidae family, a locus containing genes for lysozyme-like proteins is located near *CG3687* (in *M. domestica*, the *CG3687* gene is entirely located in the intron of one of these genes). In the basal short-horned *Hermetia illucens*, the homologue of the *CG3687* gene is also located next to *HLF* and *CG15701*, but the remaining “neighbor” genes are different: *SNRNP27*, *lyso-C-like*, and *FANCI*.

According to multiple alignments of CG3687 sequences of different *Drosophila* species the amino acid composition of the N-terminal domain of CG3687 is conserved, while the structure of the C-terminal region is very variable [65]. In the basal species *D. busckii*, the “tail” of the LysM domain is enriched in charged amino acids and contains the GSISSA motif. This structure of the C-terminal region may be close to the original one for Schizophora (flies of the *Tephritidae* family have a long S/T-rich motif surrounded by basic amino acids). Many species of the subgenus *Drosophila*, as well as some species of the subgenus *Sophophora* (*D. willistoni*), lack the S/T-rich sequence, but enrichment in charged amino acids may be retained. In species of the obscura, ananassae, and melanogaster groups, the C-terminal “tail” is depleted in basic amino acids (more hydrophobic) and includes an S/T-rich sequence in which phosphorylation sites are found, as predicted by the Motif Scan program [66]. 

According to the predictions of the DNAPred program [67], CG3687 has DNA-binding activity. According to these predictions, this protein can function under certain conditions (for example, when activated by phosphorylation) as a transcription factor cofactor.

According to modENCODE expression data presented in the FlyBase, transcription of the *CG3687* gene is not detected at the embryonic stage of development and begins to be detected from the third instar larval stage, reaching a peak in adult males [68]. The analysis of tissue-specific expression shows that the gene has maximum expression in testicular tissues. The gene is expressed at a low level in the fat body, imaginal discs, and at a very low level in the digestive system. 

Under stress conditions, the *CG3687* gene falls into the same co-expression group with the genes for catabolism (glycolysis, the Krebs cycle) and spermatogenesis (morphogenesis of cell structures, spermatozoa motility and adhesion) [68]. The cellular localization of CG3687 is unknown; according to data on protein partners, it can be located in the cytosol, closely associated with the membranes of the ER, mitochondria, and endosomes, but it is not integrated into them. The only known fact is knockdown of the *CG3687* gene results in a flightless phenotype [61,69].

### 3.2. Gagr 

The *Gagr* gene is known only in insects and presumably arose as a result of domestication of the *gypsy* retroelement in *Drosophila* ancestors [12] (Figure 2). Most *Drosophila* species are characterized by three features of the Gagr primary structure: GN repeats in the N-terminal region, a transmembrane domain or at least a GV-rich region, and a serine-enriched C-terminus. 

The function of the GN repeats is not clear. The GV-rich region, if sufficiently long, can function as a transmembrane domain that anchors the Gagr protein in the ER membrane [12]. The C-terminus probably remains in the cytosol and may be involved in protein–protein interactions, such as serine phosphorylation. The C-terminus of Gagr is also not conserved but is almost always a sequence enriched in serine, lysine, asparagine/glutamine, and tyrosine (but in species of the melanogaster group and *D. ananassae*, the motif (YN/NY)(K/Q)(G/S/T)3-5 is more common, and closely grouped lysines are less common), and it is highly likely to contain phosphorylation sites. In addition, the Motif Scan program predicts six N-glycosylation sites for asparagine in the C-terminal part of the protein. Many membrane proteins undergo glycosylation. N-glycans assist protein folding and disulfide bond formation in the ER by serving as recognition or sorting signals, allowing glycoproteins to interact with a variety of chaperones [70,71,72].

According to the data of a large-scale transcriptomic analysis of gene expression during individual development, *Gagr* gene expression was detected at the embryonic developmental stage 13–16 (60–180 min after laying) in embryonic enocytes, embryonic hindgut, and embryonic/larval corpus cardiacum [73].

According to another large-scale analysis of transcriptomes of various tissues in larvae and adults, *Gagr* expression has been observed at the highest level in the tissues of the hindgut in larvae and adults, and *Gagr* expression has been observed at a lower level in the fat body and head of adults [68]. According to the data of mass spectroscopy in adults, *Gagr* was found in the proteome of spermatozoon (the analysis was carried out in 5–7-day-old males) [74].

## 4. Evolution of Gagr and Its Partners in the Context of Stress-Signaling Pathways

In the studied complex of interacting *D. melanogaster* proteins, two conserved pairs can be distinguished: Pdi/14-3-3e and CG6013/eIF3j [16]. These proteins are joined together by their co-localization in the ER.

Physical interaction between CG6013 (homolog of CCDC124) and eIF3j has been shown only for *Drosophila*. However, for both yeast and humans, interactions have been suggested based on co-expression profiles and on the basis that yeast and human CCDC124 homologues exhibit ribosome-binding activity [54,55]. The direct interaction of proteins 14-3-3e and Pdi has also been experimentally confirmed only in *Drosophila* [16]. There is no experimental evidence for other organisms. However, human proteins P4HB (Pdi homologue), CCDC124, and eIF3J are components of the stress granule, which includes 260 proteins [75]. The stress granule does not contain the 14-3-3e homologue, a YWHAE protein. However, it can interact with P4HB prior to the formation of a stress granule.

Apparently, the complex of CCDC124 and eIF3j proteins is the most conserved component of the studied system of interacting proteins. Together with TFs, they form a signaling pathway capable of influencing the functioning of ribosomes at all stages from maturation to translation to termination [55]. On the way to evolution from yeast to mammals, new partners from other signaling pathways, as well as adapters, are added to the conserved CCDC124/eIF3j pair, which makes it possible to localize the components of the resulting multiprotein complex on membranes or inside organelles [55,75]. In different taxa of animals, this process occurred independently, so that the composition and nature of the interaction of components within the complex are different for them.

**Figure 3:**
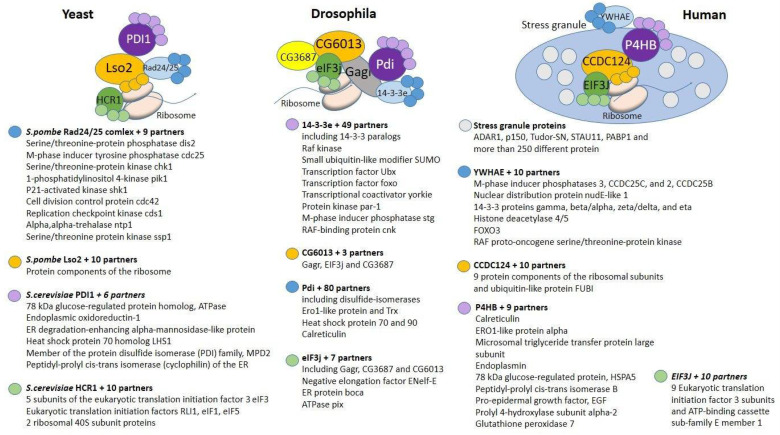
The structure of protein–protein interactions obtained experimentally for yeast, *Drosophila* and human (according to the STRING database [76], and after [16] for *Drosophila*). Homologous proteins are marked with the same color.

In yeast, the components of the complex function independently without physically interacting [35,51,54], while in mammals, such conjugation is achieved by the formation of stress granules (Figure 3). In *Drosophila*, the *Gagr* protein acts as a link and is an adapter that couples the functions of CCDC124/eIF3j (regulation of ribosome biogenesis and translation [45,46,47,48,49,54,55]) with the functions of 14-3-3e/Pdi (redox signaling [34,43]).

According to our data, the *Gagr* gene in *Drosophilidae* evolved by acquiring binding sites for the transcription factors kayak and STAT, the activity of which is modulated by the JNK and JAK/Stat stress pathways [13]. As we have shown in the study, the *Gagr* gene is significantly activated by oxidative stress. However, after a recovery period (12 h), its expression becomes comparable to the basal level. These data indicate that *Gagr* function is necessary during stress development to a greater extent than during the recovery period. At least in *D. melanogaster*, the *Gagr* gene is closely integrated into the regulatory network of signaling cascades: its transcription depends on signals from the JNK and Jak-STAT pathways.

As we have shown [13], the binding motif for the JNK pathway transcription factor kayak is present in the promoter region of the *Gagr* homologs of Sophophora and *Drosophila* species. In addition, there is a binding motif for Stat92E in the melanogaster subgroup, but it is absent in *D. ananassae*. Finally, the second Stat92E motif is found only in a group of related species: *D. melanogaster*, *D. simulans*, and *D. sechellia*. Thus, based on an analysis of potential regulators of stress-induced activation of *Gagr* and a search for appropriate motifs in various *Drosphila* species, we conclude that Stat92E and kayak are the main candidates for activating *Gagr* under stress.

The assumption of this regulation is in good agreement with the results of our experiments and other studies in which the activation of *Gagr* expression is observed in response to significant stress (viral infection, oxidative stress caused by peroxo compounds) [14,15]. The JNK signaling pathway has many functions, regulating a diversity of processes from cell movement during embryogenesis to the stress response of cells after environmental insults. In both *Drosophila* and higher organisms, JNK takes part in different processes, including apoptosis, proliferation, differentiation, cell migration, tumorigenesis, cell competition, and processes of cell regeneration [77,78]. The kayak protein (a part of the AP-1 transcription factor) is a developmentally regulated TF that may play a role in the function or determination of a particular subset of cells in a developing embryo [79]. It is able to carry out its function either independently of or in conjunction with other TFs [79]. In wounded tissues, JNK is activated in the damaged cells to ensure their apoptotic death and in the surviving cells to promote their cellular reprogramming and proliferation [77].

JNK and Jak/STAT activation in imago promote the proliferation of stem cells (SCs) in response to oxidative or ER stress and infection [78]. In addition, the JNK pathway regulates *upd3* (effector of Jak/STAT pathway) expression, which is necessary for optimal renewal of the intestinal epithelium and survival following septic injury [80]. JNK also becomes widely activated in the intestinal epithelium of aging flies, inducing excessive proliferation of ISCs [81]. In addition, autophagy plays a role in *Drosophila* ISCs to maintain proliferation and preserve the stem cell pool.

Considering that *Gagr* is expressed in oenocytes (cells of the fat body with immune functions, similar to hepatocytes), in the tissues of the hindgut in larvae and adults, in the intestinal region necessary for stress-induced repair [81], and in the testes, it can be concluded that that the expression of the *Gagr* gene is observed in imago tissues with a high potential for stress-induced proliferative activity. Thus, *Gagr* likely participates in the control of morphogenesis at the embryonic stage of development, and in adults, in post-stress tissue regeneration. Switching off the *Gagr* gene does not impair the viability of flies, but it can affect their survival under stressful conditions.

The function of the complex may also be to regulate the proliferative activity of the testes. Spermatogenesis originates from spermatogonial stem cells (SSCs). Increased JNK activity promotes SSCs proliferation [82]. Additional roles of the JNK pathway in testis have been found during episodes of stress. The JNK pathway underlies cellular plasticity in the testis, enabling survival of resident stem cells during stress and inducing cell reprogramming to replenish stem cell pools once the stress is terminated [83].

We have shown that the protein product of the *Gagr* gene has membrane localization [12]. Based on the proven localization of some of its partners, we can conclude that it is inserted into the ER membrane and binds to a component of the translation system and signaling proteins. This indicates the formation of a novel function of the domesticated *gag* gene in the *D.melanogaster* genome. 

**Figure 4:**
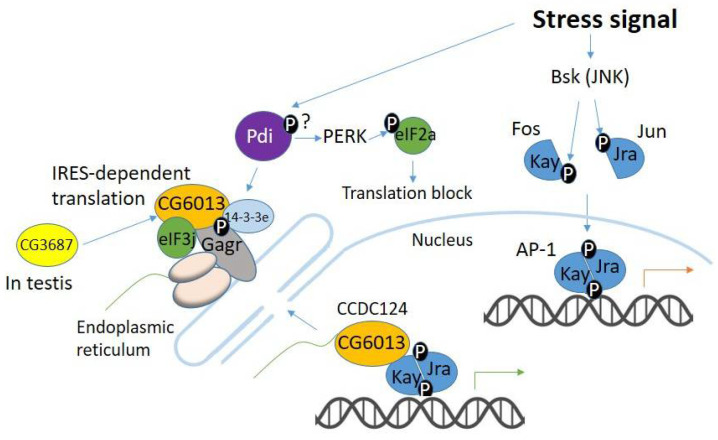
Proposed scheme of stress-induced activation and operation of the complex, including the Gagr protein. JNK activation in imago promotes response to oxidative or endoplasmic reticulum (ER) stress and infection. Pdi, as a redox-sensitive chaperone, receives and transmits the signal to its partners—14-3-3e and Gagr. At the same time Pdi induces the PERK protein to block canonic translation. The activity of both Pdi and Gagr proteins can be modulated by phosphorylation. 14-3-3e is able to bind to phosphorylated partners, including Gagr, which binds to CG6013 (CCDC124 homolog) and eIF3j. The Gagr protein is anchored in the ER membrane and may serve as an adaptor protein coordinating its partners’ interaction. The function of Gagr may also be to recruit resting ribosomes to the ER for their CG6013(CCDC124)-dependent reactivation. CG6013 (as its yeast homolog) can enter the nucleus and stimulate transcription of a specific set of genes; after that, it can carry mRNAs to the site of translation with the participation of translation factor eIF3j. In testis, CG6013 can presumably be activated by CG3687, which may be a partner of TF(s) involved in spermatogenesis.

In Figure 4, a diagram of a possible scenario for the functioning of the complex in *D. melanogaster* is shown. Under oxidative stress, Pdi, as a redox-sensitive chaperone, is the first to perceive the signal and transmit it to partners [34]. The activity of Pdi and Gagr proteins can be modulated by phosphorylation, and 14-3-3e is able to bind to phosphorylated partners [19,41]. In parallel, 14-3-3e can activate the activity of the Ras/MAPK pathway [17]. In turn, Gagr (possibly through a partnership with 14-3-3) binds to CG6013 and eIF3j, as well as a set of mRNAs to the ribosome. Unfortunately, the *CG6013* gene is poorly studied. There are no research papers aimed at studying gene function. As such, we can assume the function based on homology with CCDC124 and Lso2 proteins. The function of Gagr may also include recruiting resting ribosomes to the ER for their CCDC124-dependent reactivation, as occurs in the yeast *S. cerevisiae* with the participation of the Lso2 protein (but without coupling to the ER). It is possible that in Gagr it causes an alternative pathway for ribosome attachment to the translocon and an alternative pathway for translation under stress conditions when normal protein synthesis is impossible.

Considering the similarity of CG6013 both to the yeast Oxs1 and Lso2 proteins and to the human CCDC124 protein, it can be assumed that CG6013 is involved in a signaling pathway similar to Pap1/Oxs1 [51,55]. In *D. melanogaster*, this pathway probably involves ribosomes and the apparatus responsible for protein synthesis in the ER. It is possible that the CG6013 protein is an important signaling molecule circulating between the translation apparatus and the nucleus. Its nuclear functions can be activation or suppression of the expression of some genes by modulating the activity of TFs (possibly, Jra), binding of transcripts of these genes, their processing, and export [51,56]. Possible functions associated with translation include directing transcripts to the ER, initiation (together with Gagr and eIF3j) of a non-canonical IRES-dependent translation pathway, and reactivation of resting ribosomes. Apparently, CG3687 can be activated in testicular tissues by binding to CG6013 (having an elevated level of expression in the testes), which can presumably be a partner of TFs involved in the processes of spermatogenesis (morphogenesis of cell structures, motility and adhesion of spermatozoa).

## 5. Conclusions

During the phylogenesis of the genus *Drosophila*, the *Gagr* gene arose and underwent significant changes: the regulation of the promoter region of the gene became more complicated, and its protein product acquired a transmembrane domain. The result of these transformations was the inclusion of this gene in the signaling pathway controlled by stress cascades. It seems that the *D. melanogaster* Gagr protein is anchored in the ER membrane and may serve as an adaptor protein coordinating its partners’ interaction. We can’t say without experimental verification about the role of the Gagr protein in the complex. On the one hand, it may serve as a chaperone to help the cell survive under strong insult conditions. On the other hand, it can mediate IRES-dependent translation and promote proliferation under mild insult or stress conditions. These hypotheses require further verification. Finally, we cannot say definitely how this complex functions without Gagr in other insects because homologous proteins of the complex are not studied in other insects. And this is also the subject of further research.
